# Use of intensity modulation for missing tissue compensation of pediatric spinal fields

**DOI:** 10.1120/jacmp.v4i4.2497

**Published:** 2003-09-01

**Authors:** M. H. Phillips, P. S. Cho, H. Parsai, J. G. Douglas

**Affiliations:** ^1^ Department of Radiation Oncology University of Washington Medical Center Box 356043 Seattle Washington 98195‐6043

**Keywords:** medulloblastoma, compensators, intensity‐modulation

## Abstract

Irradiation of the cranio‐spinal axis is often one of the treatment modalities of certain childhood cancers, e.g., medulloblastoma. In order to achieve a uniform dose to the spinal cord, missing tissue compensators are required. In the past, our practice was to fabricate compensators out of strips of lead. We report on the use of intensity modulated fields to achieve the desired compensation. Seven cases of pediatric cancer whose treatment involved irradiation of the cranio‐spinal axis had compensators designed using a beam intensity modulation method rather than making mechanical compensators. The compensators only adjusted for missing tissue along the spinal axis. Comparisons between calculated and measured doses were made at depth in phantoms and on the surface of the patient. The intensity modulated fields were delivered using a step‐and‐shoot delivery on an Elekta SL20 accelerator equipped with multileaf collimator. The intensity‐modulated compensators provided more flexibility in design than the physical compensator method. Finer intensity steps were achievable, more accurate dose distributions were able to be calculated, and adjustments during treatment, e.g., junction changes, were more easily implemented. Convolution/superposition dose calculations were within ±3% of measurements. Intensity modulated fields are a practical and more efficient method of delivering uniform doses to the spine in pediatric cancer treatments. They provide many advantages over mechanical compensators with regard to time and flexibility.

PACS number(s): 87.53.–j, 87.90.+y

## I. INTRODUCTION

Cancer of the central nervous system (CNS) accounts for 18% of all childhood cancers.^1^ ^,^ ^2^ Medulloblastoma is the most frequently occurring CNS tumor in children under the age of 18 and accounts for approximately 29% of all primary CNS tumors of childhood.^2^ Treatment strategies have evolved over the past two to three decades with cranial spinal irradiation as the backbone of all approaches. Recent studies have demonstrated the efficacy of craniospinal irradiation to a reduced dose of 2340 cGy and chemotherapy for average risk patients.^3^

Techniques involved with the treatment of the cranio‐spinal axis generally employ a single posterior field^4^ which results in dose inhomogeneities of approximately 5–15% along the length of the spine. In order to achieve a more homogeneous distribution, a missing tissue compensator must be used. Given the anatomy, the tissue compensation is only required along one dimension: the cranio‐caudal axis.

In the past, it was our practice to fabricate a compensator using strips of lead. Due to the difficulty of designing these types of compensators in the treatment planning system, typically three to five intensity levels were chosen, determined by the thickness of the lead strips. This system was less than optimal for several reasons. In the treatment planning system, the design of the compensators was modelled by a series of blocks stacked on top of one another; the block design was by trial‐and‐error. The time needed for this process and the fixed thickness of lead strips limited the number and resolution of intensity steps that were used. Once designed, the dose calculation was not very accurate off‐axis due to the complicated nature of the scatter arising from the absorbing compensator filter. The time needed to make the compensator made it difficult to modify once designed and fabricated. The placement of the compensator on the block tray also blocked the light field, thereby adding steps in the alignment and setup procedures.

The capability of delivering step‐and‐shoot intensity modulated fields on our accelerator provided us with a better solution to the tissue compensation problem. A number of publications have described procedures for using intensity modulated compensators, particularly for breast tangent fields.^5^ ^–^ ^9^ Cranio‐spinal irradiation is a simpler case since the modulation is only one‐dimensional, resulting in simpler inverse planning algorithms, delivery sequences, and quality assurance methods. In this paper, we report our implementation of such a system and the results in a small series of patients.

## II. METHODS AND MATERIALS

The intensity modulated compensator (IM‐compensator) was implemented using an in‐house treatment planning system, Prism.^10^ Results are presented for seven patients. Two dose calculation algorithms were used: (a) a broad beam model utilizing output factors, tissue phantom ratios, and off‐axis ratios,^11^ ^,^ ^12^ and (b) a superposition/convolution model,^13^ ^,^ ^14^ which utilizes 1 cm×1 cm pencils and empirically derived values for kernels, absorption, and head scattering. The two algorithms were applied as a comparison given that the IM compensator technique is relatively simple, could be implemented in older treatment planning systems without pencil beam algorithms. The fields were transferred to an Elekta (Elekta Oncology Systems, Crawley, England) SL20 linac using the Dicom‐RT protocol.

**Figure 1:**
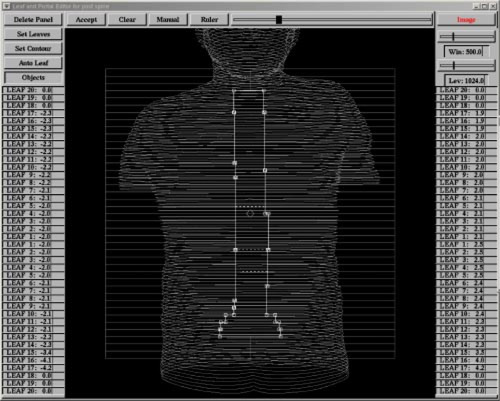
(Color) Screen shot of the beam portal panel showing in solid white lines the open beam portal overlaid on a wire frame image of the surface of the patient. Also shown in dotted white lines are the superior borders of the segments that irradiate the inferior part of the volume. The MLC leaves are shown in light gray.

Compensator design was achieved in several steps: (1) anatomy delineation and dose point designation, (2) portal design, (3) “inverse planning,” and (4) leaf sequencing. Step 1 is the same as in any 3D conformal process in which all of the relevant anatomy is contoured, e.g., skin, spinal cord, and vertebrae. CT images were obtained with the patient lying in a prone position with a specially designed head holder to allow for anesthesia. Dose points were placed along the anterior aspect of the spinal cord and designated the locations at which a homogeneous dose was desired. Step 2 is another standard process in which a field is designed that encompasses the entire spinal region (Fig. 1). The multileaf collimator (MLC) was oriented so that the direction of leaf travel was perpendicular to the spinal axis. This meant that each leaf pair of the MLC covered or uncovered a 1 cm length of spine. With the Elekta accelerator, the top and bottom edges of the fields did not have to end at an integer number of centimeter steps, since the X diaphragms were used to set the top and bottom field edges.

Step 3 involved a very simplified version of an inverse planning algorithm. Once the isocenter and SSD were set, the patient was divided into narrow slabs corresponding to the shadow of an MLC leaf pair. Dose points were interpolated between the user‐defined dose points and placed in the center of each of the slabs. Using one of the dose algorithms (described above), the dose per monitor unit (dose/MU) to each of the new dose points was calculated. That curve was inverted and multiplied by the prescription dose to obtain the monitor units at each point. The user was asked to define a minimum number of monitor units to be delivered in a single segment. The dose point with the minimum MU's determined the number of MU's to apply to the open field. This value was subtracted from the curve. Using the minimum MU/segment value, an iterative process found the next set of points that fell within the allowable MU's, subtracted that value from the curve, and repeated until the maximum number of MU's was delivered.

**Figure 2:**
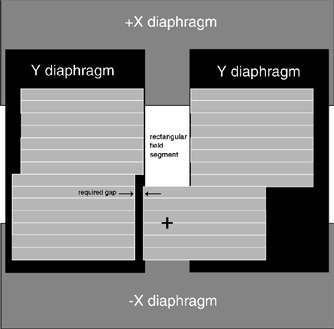
Schematic of Elekta MLC and backup diaphragms illustrating the “flagpole.” The system constraint that the top and bottom of the field be delineated by the X diaphragms requires a special approach when the entire beam portal lies either above or below the central axis. This is accomplished by opening leaves between the portal and the central axis in such a way that the Y diaphragm can cover the resultant opening.

Step 4 involved a simplified version of a leaf sequencing algorithm. Each set of contiguous points that corresponded to one of the MU delivery segments identified a set of MLC leaves. A new beam segment was created with the appropriate number of monitor units and with the designated MLC leaves open. The actual aperture that was sent to the accelerator had to meet Elekta's constraints for leaf and diaphragm positions. The most significant constraint was that the bottom and top of each field must be set by the X diaphragms and that the X diaphragms cannot cross the central axis. For the beam segments used in the spinal compensator, many apertures were either wholly above or below the central axis. This resulted in a “flagpole” field: leaves that would normally be closed between the aperture edge and the central axis were set to create a narrow slit that was lateral to the true aperture edge. This slit was then covered by the perpendicular Y diaphragm. (See Fig. 2).

The design process was completed by calculating the dose to the dosepoints and inspecting them using a built‐in spreadsheet (Table I). Since the dose/MU to each point was calculated using the open field, the actual doses delivered once the segments were created could be significantly less than desired due to differences in scatter factors between the open field and the smaller segments. The MU's could be adjusted automatically, but it was found that using the spreadsheet was more useful. By inspecting the dose delivered to each point by each segment, small changes to MU's and field shape could be implemented. Typically this would involve deleting very small fields and sometimes increasing the MU's of a larger field, or incorporating (by opening leaf pairs) one small field into a larger one. Since the beam MU's could be adjusted on the spreadsheet, the final plan was easily tailored by adjusting any segment's MU's to achieve the most homogeneous dose.

For mechanical compensators, intensity modulation was accomplished by designing blocks corresponding to strips of lead. Two thicknesses were used: 0.4 mm (0.973 transmission for the energy used, 6 MV) and 0.8 mm (0.947 transmission) were used. The number, position, and length of each strip was obtained by a trial and error procedure, starting with an initial solution computed by the exponential attenuation relationship.

Once the compensator fields were designed, a software tool was used to calculate the dose at a set of points in a phantom. A diode array (Sun Nuclear, Melbourne, FL) with solid water slabs overlaying it was used to measure the dose along the length of the field. The calculated doses at the diode locations were compared to the measured doses.

At any time, modifications could be made to the compensated field, including junction changes or monitor units. The new values were then transferred from the planning computer to the accelerator.

## III. RESULTS

Compensators have been designed for seven patients (see Table II). In all cases, the calculated compensation has produced dose distributions that were within ±4% of the prescribed dose, while most were ±2%. The number of segments for each compensator ranged from 6 to 12, with the fewer segments generally correlated to a larger minimum number of monitor units per segment, although the individual patient anatomy had a larger effect. As discussed in Sec. II, some post‐processing was done. In nearly all cases, at least one auto‐generated segment was removed, and the number of monitor units adjusted for several segments. Typically, several segments would be adjusted to two or three times the minimum number of MU's. In one case, one segment had 25 MU's. In each case, the entire time required to design the compensator and prepare it for use at the linac (after the anatomy had been entered) was less than 0.5 hr.

**Figure 3:**
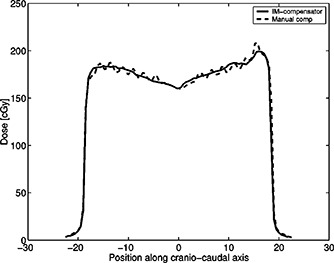
Plot of the measured doses in phantom of the IM‐compensator and the manual compensator. SSD=105 cm, depth of detectors = 5 cm.

Figure 3 illustrates the dosimetry for Patient 6. This patient had an IMRT compensator designed, but for reasons relating to patient immobilization, it was decided to use a mechanical compensator. The dosimetry was performed on both compensators.

**Table I:** A schematic version of the treatment planning spreadsheet, which shows the total dose to each dose point, and the dose contribution to each point from each segment. Doses are rounded to integer values to improve visual clarity. Dose point names refer to cervical, thoracic, and lumbar vertebrae. For clarity, the small fractions of dose contributions from each beam due to scattering are omitted, which sometimes results in the total dose not equalling the sum of the segment doses.

		Parent	Seg‐1	Seg‐2	Seg‐3	Seg‐4	Seg‐5	Seg‐6	Seg‐7
Point	$D_{total}$	223 MU	3 MU	5 MU	8 MU	15 MU	3 MU	3 MU	3 MU
C‐5	180	160	2	3	5	10			
C‐6	182	162	2	4	5	9			
C‐7	182	169	2	4	5	2			
T‐1	179	173	2	4					
T‐2	182	176	2	4					
T‐3	182	179	2						
T‐4	183	180	2						
T‐5	183	180	2						
T‐6	181	179	1						
T‐7	183	182							
T‐8	181	180							
T‐9	182	179					2		
T‐10	181	177					2	2	
T‐11	182	176					2	2	1
T‐12	180	174					2	2	2
L‐1	179	171					2	2	2
L‐2	182	175					2	2	2
L‐3	182	175					2	2	2
L‐4	181	174					2	2	2
L‐5	177	171					2	2	2

For those cases in which junction changes were required in the cervical spine region, the superior aspect of all fields bordering that aspect were modified the required amount. The resulting dose changes were reviewed and in no case was it determined that any other changes to the compensator design were needed.

**Figure 4:**
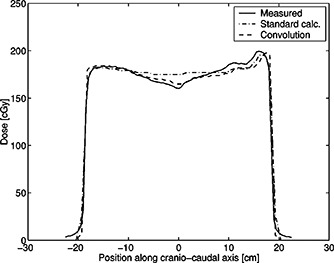
Plot of the measured doses in phantom of the IM‐compensator and the doses calculated using the standard algorithm and with the pencil beam algorithm. Maximum difference between the measured and pencil‐beam calculated values is 3.1%.

Figure 4 shows the dose along the central part of the compensator (for the same patient as in Fig. 3) as calculated using the two different algorithms described in Sec. II, and compares them with the result of the measurements.

**Table I:** Summary of the IM compensators designed for seven patients. Doses and monitor units are for a single fraction. MU=monitor units. Minimum MU's per segment is the minimum number of monitor units delivered per segment. Compensated Dose refers to the doses per fraction (and dose range) as calculated by the planning system for the IM compensator. Uncompensated dose is the dose (and dose range) for the uncompensated spinal field. (^*^) refers to a separate plan for Patient 1 with a different value for minimum MU's per segment.

	Number of segments	Minimum MU's per segment	Compensated dose (cGy)	Uncompensated dose (cGy)
1	11	3	180±4	180 +21, −15
1*	9	5	180±4	
2	8	3	180±4	180 +8, −30
3	7	5	180 +7, −5	180 +6, −33
4	6	6	180±3	180 +4, −18
5	8	5	180±4	180 +7, −28
6	9	3	180±3	180 +12, −12
7	12	5	180±4	

## IV. DISCUSSION

The major reasons for implementing the IM‐compensators were for increased efficiency and flexibility. The time required to design and prepare the compensators was reduced from several hours to less than one hour. The increased flexibility was an important advantage in several ways. With regards to junction changes, it was possible to modify the scheduled junction changes after treatment started since the size of the compensator was adjustable. Although we have not needed to adjust the modulation because of junction changes, this was also very easy compared with the mechanical compensators. Finally, it was sometimes the case that a patient was started on treatment before the compensator was ready. Due to the ease of using the spreadsheet, it was trivial to account for unmodulated fractions already delivered. It might be pointed out that this boon will be reduced in importance since one of the major factors in starting treatment without the physical compensator was the long time required to produce it.

It was found that patient geometry was the largest factor in the details of the IM‐compensator design. This affected the number, shape, and position of the segments, and the number of monitor units for each segment. Three monitor units were chosen as the minimum to be delivered in a segment, but five or six MU's also resulted in acceptable dose homogeneity. In each case, the monitor units of a few segments were adjusted. The magnitude of the adjustment depended on the anatomy, with most adjustments resulting in 10 MU's or less, but in two cases, 15 and 25 MU's were used.

Segment sizes were also very anatomy‐dependent. The largest modulation was always in the superior and inferior regions. In some patients the depth gradient was quite steep resulting in three or four segments, each differing from the preceding segment by one pair of closed leaves. In others, the gradient was much less steep so that sequential segments were quite different in size.

DICOM‐RT was used to transfer the fields from Prism to the Elekta SL20 linac. The speed and ease of the system made modifications easy to handle. The time needed for treatment was an issue since many of these patients need to be anesthetized. On our system, each segment added approximately 5 to 10 sec., so that it took only about 1 minute longer to deliver the radiation. Typically, the delivery sequence was set up so that the first segment was the open field that encompassed the entire volume, then the superior segments were delivered from largest to smallest, and then the inferior segments.

The dose calculation algorithm was seen to be significant, typically 10 cGy/fraction on central axis. The TPR algorithm, which uses an equivalent square approach, gives too much weight to field elements far from the central axis, resulting in a predicted dose that is higher than measured near the center. The pencil beam algorithm, which correctly accounts for scatter from each pixel in the field, is much more accurate.

Interleaf leakage in “flagpole” fields was not considered to be a problem for two reasons. One, the narrow extent of the leakage gets averaged out during the course of treatment due to the magnitude of setup errors. The second is that the flagpole fields contribute only a relatively small fraction of the total delivered dose.

One potential disadvantage with the IM‐compensator method is related to treatment interruptions. Although the Elekta control system has a facility for completing interrupted treatments, there may be the possibility that the patient will move or be set up in a slightly different position, thereby creating a region that would be over‐ or under‐dosed. In addition, the procedure to recover from the interruption can be time‐consuming. For this reason, in one case in which the patient was not anesthetized and exhibited behavioral problems, it was decided to use a mechanical compensator for ease of use by the therapists.

## V. CONCLUSION

The intensity modulated compensator method proved to be a very efficient and flexible technique for spinal irradiation. The time required to design and fabricate the compensator was greatly reduced. Flexibility in changing or adapting the treatment was also greatly improved. A convolution/superposition algorithm provided dose calculations with 3% of the measured values in a phantom.

## ACKNOWLEDGMENT

This work was partially supported by a grant from Elekta Oncology Systems.
